# Reversal of resistance to chemotherapy following anti-programmed cell death-1 immunotherapy in metastatic lung adenocarcinoma: A case report

**DOI:** 10.1097/MD.0000000000013427

**Published:** 2018-12-10

**Authors:** Xiaoli Liu, Huaimin Liu, Suxia Luo

**Affiliations:** Affiliated Cancer Hospital of Zhengzhou University & Henan Cancer Hospital, Zhengzhou, Henan, China.

**Keywords:** chemotherapy, metastatic lung cancer, programmed cell death-1

## Abstract

**Rationale:**

For metastatic non-small cell lung cancer with no epidermal growth factor receptor mutations or anaplastic lymphoma kinase gene rearrangements, programmed cell death-1 (PD-1) blockade is preferentially recommended post first-line chemotherapy. However, still many patients do not respond to these agents. After development of resistance to PD-1 blockade, further evaluation of chemotherapy regimen will be necessary.

**Patient concerns:**

A 57-year old man had cough with minimal whitish expectoration. Computed tomography (CT) scans showed that he had an upper lobe mass of his left lung and multiple lymphadenectasis, including mediastinal and hilar lymph nodes, and also to the right intrapulmonary lymph nodes.

**Diagnoses:**

The patient was diagnosed with adenocarcinoma after a biopsy was conducted on the upper lobe mass of his left lung.

**Interventions:**

The patient received pemetrexed plus cisplatin (Pem-Cis) treatment for 6 cycles and sequential thoracic radiation as a therapeutic schedule. CT demonstrated a confirmed partial response after these treatments. Three months later, the tumors continued to grow. The patient received successive pemetrexed-based chemotherapy regimens; however, these regimens failed to stop tumor progression. The patient subsequently underwent 6 cycles of PD-1 mAb pembrolizumab treatment.

**Outcomes:**

Sensitivity of chemotherapy was restored, and the patient displayed a reduction in the size of enlarged mediastinal and hilar lymph nodes after 2 cycles of treatment with Pem-Cis, the initially used chemotherapy regimen.

**Lessons:**

This outcome suggests that PD-1 blockade holds promise as a treatment strategy for reversion of chemotherapy resistance in refractory lung adenocarcinoma and warrants additional studies.

## 1 Introduction

For patients with advanced non-squamous non-small cell lung cancer (NSCLC) who lack sensitizing epidermal growth factor receptor (EGFR) mutations or anaplastic lymphoma kinase (ALK) gene rearrangements, platinum-based doublet chemotherapy regimens, such as pemetrexed plus cisplatin (Pem-Cis), are currently recommended.^[1]^ Superior efficacy and reduced toxicity has been reported for Pem-Cis, in comparison to other chemotherapy regimens. While a small number of patients initially respond well to this chemotherapy regimen, the majority of patients ultimately develop drug resistance to this regime.^[1]^ For subsequent systemic therapy, immune checkpoint inhibitors such as anti-PD-1 mAb are now recommended as preferred agent due to improved overall survival rates, longer duration of response, and fewer adverse events compared with conventional cytotoxic chemotherapy.^[2–4]^ For patients progressing beyond these second-line and third-line treatments, switching to an alternative chemotherapeutic agent is commonly selected, as the initially used chemotherapy regimen is often not effective for refractory tumors.^[1]^ Here, we present 1 case of a patient diagnosed with NSCLC, for whom initial chemotherapy resistance was successfully reversed after blocking PD-1 via pembrolizumab.

## 2 Case report

**Figure 1:**
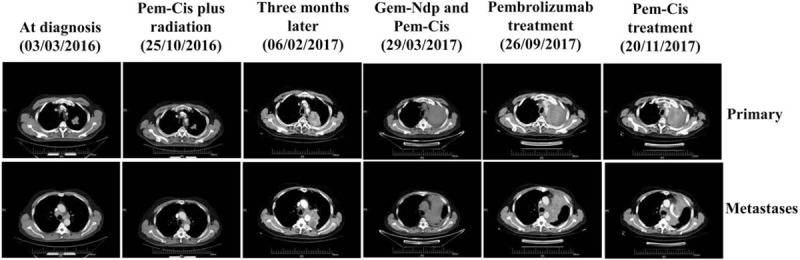
The patient exhibited a partial response after 6 cycles of treatment with Pem-Cis chemotherapy and sequential thoracic radiation. Three months later, the tumor grew back, and the patient received both Gem-Ndp and Pem-Cis chemotherapy regimens. The tumor continued to grow, and the patient was subsequently treated with pembrolizumab; however, no tumor shrinkage was observed. Interestingly, following this treatment the patient was sensitive again to Pem-Cis, as lymph node metastases shrunk after 2 cycles of initially chemotherapeutic agents was used (as of December 2, 2017).

A 57-year-old man with cough and minimal whitish expectoration was diagnosed with adenocarcinoma after a biopsy was conducted on the upper lobe mass of his left lung on March 4, 2016. Mutational analysis revealed that he lacked EGFR mutations or ALK gene rearrangements. CT scans showed that this patient had developed multiple metastases, including mediastinal and hilar lymph nodes, and also to the right intrapulmonary lymph nodes. He received 6 cycles of Pem-Cis chemotherapy, followed by thoracic radiation. Review of the CT scan at completion of sequential chemoradiotherapy treatment resulted in a significant shrinkage in the primary tumor in his left lung, while simultaneously resulting in a slight increase in metastatic lymph nodes (Fig. 1). In February 6, 2017, CT scans showed that the tumor progressed rapidly. He was then scheduled to receive gemcitabine plus nedaplatin (Gem-Ndp) chemotherapy. However, the patient had to discontinue the planned 2nd cycle of this regimen as he developed moderate pneumonia. After systemic antibiotic treatment, the patient improved symptomatically and became increasingly energetic. However, CT scans on March 29 revealed that his tumors had progressed further. Owing to his poor physical condition after Gem-Ndp treatment, the chemotherapy regimen was changed to Pem-Cis. However, CT scans on May 11 failed to detect any shrinkage in his tumor. In addition, lymph node metastases increased post-treatment. The patient subsequently started standard of care pembrolizumab treatment at 2 mg/kg intravenously every 3 weeks for 6 cycles. During hospitalization, the general condition of the patient was good, with signs of fatigue only present at the first 2 days after each pembrolizumab infusion. The treatment failed to result in an anti-tumor response. The patient then received 2 cycles of initially used chemotherapy regimen Pem-Cis. Interestingly, we detected a significant shrinkage in the enlarged mediastinal and hilar lymph node metastases, with the primary site in the left lung exhibiting no further progression.

**Figure 2:**
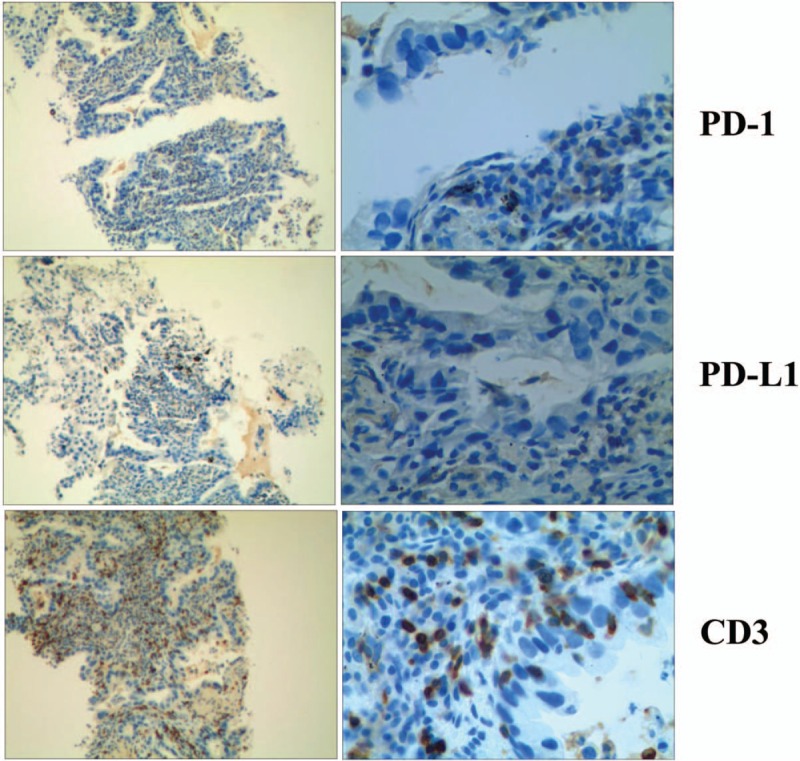
Immunohistochemical analysis of a pretreatment tumor from the patient showed CD3^+^ T cell infiltration, but no PD-1 or PD-L1 expression (magnification: left panel, 10×; right panel, 40×).

Recent studies have suggested that clinical responses are associated with several potential biomarkers, including PD-1, PD-L1, and CD3.^[5]^ To investigate the clinical association of these factors with the efficacy of PD-1 blockade, we performed immunohistochemistry analysis of PD-1, PD-L1, and CD3 in specimens obtained from this patient. The results showed that the tumors from exhibited CD3^+^ T cell infiltration, but no PD-1 or PD-L1 expression (Fig. 2).

## 3 Discussion

Although 2 recent phase III trials (Keynote 189 and IMPOWER 150) showed that standard chemotherapy plus a checkpoint inhibitor extended progression-free survival and overall survival compared with chemotherapy alone,^[6,7]^ pemetrexed-based chemotherapy regimen remains as one of first-line treatments for patients with NSCLC who lack sensitizing EGFR mutations or ALK rearrangement. For patients who have progressed on or after pemetrexed-based chemotherapy, recommended subsequent systemic therapy options include immunotherapy or other chemotherapy regimens.^[1]^ However, the role of further-line treatment is controversial when therapy with immune checkpoint inhibitors failed to control tumors. Results from this case report showed that PD-1 blockade treatment reversed resistance to original chemotherapy, indicating that the initial cytotoxic chemotherapy regimen that used for first-line treatment can be re-selected for patients who progress on second-line immune checkpoint inhibitors.

We analyzed the subsets of immune cells in the peripheral blood of the patient. The baseline levels of the percentage of the PD-1^+^ subpopulation among circulating CD3^+^ T cells were much higher than those in healthy people. In addition, following treatment, this population of cells decreased slightly from 23.1% to 14.6% (Table 1). The second cell subset the patient and healthy people differed in CD56^+^ natural killer cells. It was reported earlier that in patients with regressing melanoma the population of peripheral blood cells expressing CD56 increased following PD-1 blockade treatment.^[8]^ In our study, no increase in the total number of CD56^+^ cells was observed after PD-1 blockade treatment (Table 1). Whether the PD-1^+^ subpopulation among circulating CD3^+^ T cells or CD56^+^ natural killer cells represents an appropriate biomarker on the basis of which NSCLC patients are selected for PD-1 blockade therapy remains to be resolved.

**Table 1:**
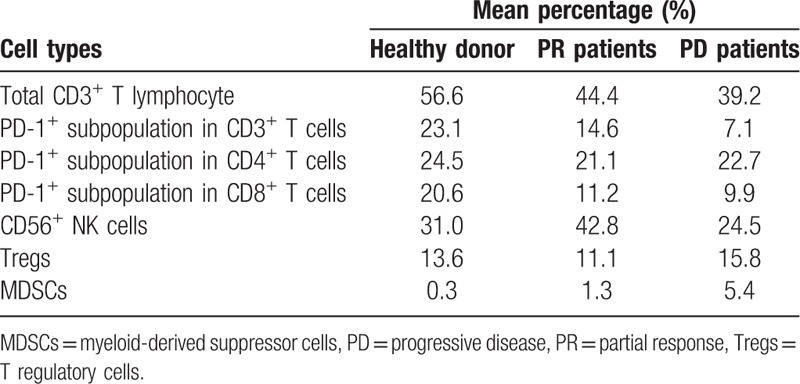
Percentage of subpopulation in the peripheral blood mononuclear cells of patients as well as healthy donors.

Because this is a report of a single patient, the results presented here are purely descriptive rendering any definitive conclusions about the impact of anti-PD-1 mAb on chemotherapy resistance challenging. However, this case illustrates that anti-PD-1 treatment in NSCLC patients with no EGFR mutations or ALK rearrangement has the potential to restore sensitivity to a chemotherapy regimen. The underlying mechanisms deserve further investigation.

## Acknowledgments

The authors thank Dr. Torsten Juelich for linguistic assistance during the preparation of this manuscript.

## Author contributions

XL Liu and SX Luo have designed the paper; HM Liu managed the patient's care in our medical oncology clinic; XL Liu and SX Luo wrote, reviewed, and revised the paper; all the coauthors revised paper critically and gave final approval of this version for publishing.

**Supervision:** Suxia Luo.

**Writing – original draft:** Xiaolli Liu.

**Writing – review & editing:** Huaimin Liu.
